# Adverse Drug Reaction Related Post Detecting Using Sentiment Feature

**Published:** 2018-06

**Authors:** Jingfang LIU, Xiaoyan JIANG, Qiangyuan CHEN, Mei SONG, Jia LI

**Affiliations:** 1. Dept. of Information Management, School of Management, Shanghai University, Shanghai 200444, China; 2. Dept. of Economics, School of Economics, Shanghai University, Shanghai 200444, China; 3. Dept. of Software Engineering, School of Smart Education, Jiangsu Normal University, Xuzhou 221116, China; 4. Dept. of Management Science and Engineering, School of Business, East China University of Science and Technology, Shanghai 200237, China

**Keywords:** Adverse drug reaction, Post, Sentiment feature

## Abstract

**Background:**

The posts related to Adverse Drug Reaction (ADR) on social websites are believed to be valuable resource for post-marketing drug surveillance. Beyond domain feature, the aim of this study was to find a more effective method to detect ADR related post.

**Methods:**

We conducted experiment on posts using sentiment features from March 8 to May 20 in 2016 in Shanghai of China. Firstly, the diabetes posts were collected; the 1814 posts were annotated by hand. Secondly, sentiment features set were generated and the *χ*^2^ (CHI) statistics were used to select feature. Finally, we evaluated the effectiveness of our method using the different feature sets.

**Results:**

By comparing the posts detection performance of different feature sets, using sentiment features by CHI statistics can improve ADR related post detection performance. By comparing the ADR-related group with the non-ADR group, performance of ADR related post detection was better than the performance of non-ADR post detection. We could obtain highest performance owing to introducing sentiment feature and using CHI feature selection technique, and the method was proved to be effective during detecting post related to ADR.

**Conclusion:**

By using sentiment feature and CHI feature selection technique, we can get an effective method to detect post related to ADR.

## Introduction

The adverse drug reaction (ADR) has always been major global health concern (1). Most common and serious ADRs were officially published in the drug labels and official platform such as Drugs@FDA. However, because controlled clinical trials have some disadvantages such as small population size and short duration, it is difficult to find out all ADRs. Oppositely, some additional adverse reactions were observed after the drugs were approved. Therefore, post-marketing drug surveillance has played a very important part in supervising ADRs. The drug manufacturers are ethically committed and legally obliged to report adverse reactions to the approved drugs.

Meanwhile, drug regulatory agencies also encourage individuals such as patients and doctors to spontaneously report the ADRs that they have ever experienced. Many health organizations have maintained post-marketing surveillance systems that enable individuals to report ADRs, such as AERS (Adverse Event Reporting System) by FDA and the VigiBase of WHO. These spontaneous reporting systems typically received tens of thousands of reports related to ADRs each year.

However, some patients experienced ADRs actually will not actively report ADR to these drug regulatory agencies because of distrust, embarrassment, inconvenient use of these systems, etc. Instead, they often turn to some informal networks to discuss their experience with ADRs such as social media services on the Internet (2, 3). Patients prefer to share and discuss the experience related to ADR in social media websites, such as https://www.askapatient.com/ and https://www.webmd.com/ (4, 5). A lot of posts related to ADR aggregated to be some communities (6, 7). These posts are believed to be valuable resource for post-marketing drug surveillance (8, 9). However, it is very time consuming and expensive to detect post related to ADR manually from a large scale unstructured posts in social website.

Some studies used text classification technology to identify post related to ADR from social website (10). These studies focused on automatic identification information related to ADR from social media (11, 12). In text classification process, several domain-specific feature sets were used to identify information related to ADR (13, 14), such as coding symbols for thesaurus of adverse reaction terms (COSTART) (15), Side Effect Resource (SIDER) (16), MedEffect and Consumer Health Vocabulary (CHV) (17). Patients usually give positive or negative opinions regarding particular drugs in their posts. If the drugs could help them much, they will have a positive attitude towards the drugs. Otherwise, if the drugs have serious side effects, people will express negative feelings about the drugs in posts (18, 19). Therefore, beyond domain features, the sentiment feature could be introduced to detect post related to ADR, and it will be considered to be helpful to improve identification performance of ADRs.

The aim of this study was to create an effective method to find and identify that whether the post was related to ADR using sentiment features.

## Methods

### Posts acquisition and annotation

In medical social media, patients have released a lot of posts. The posts content includes many aspects, such as disease condition description, seeking medical help, seeking emotional support, medications. There are a lot of posts related to ADR, which are very important for drug supervision. The aim of this paper was to use sentiment features to detect and identify that whether the post was related to ADR. The diabetic community in webmd.com has rapid growth of posts number, and the ADR of anti-diabetic drug was discussed in diabetic community. Therefore, we chose the diabetes community as the data source. Some of Antidiabetic drugs adverse effects depend on nutritional habits and combination of drugs. This question is beyond this research. The aim of this paper was to create an effective method to find and identify that whether the post was related to ADR using sentiment features. In the future research, we will try to find another method to judge that the reason of ADR is nutritional habits or combination of drugs.

Then we randomly selected 1814 posts from the diabetic community and manually annotated the posts based on whether ADRs were mentioned or not from March 8 to May 20 in 2016 in Shanghai of China. These posts were independently annotated by two experts in ADR research. Each post was annotated based on whether ADRs are mentioned or not.

**Fig. 1:**
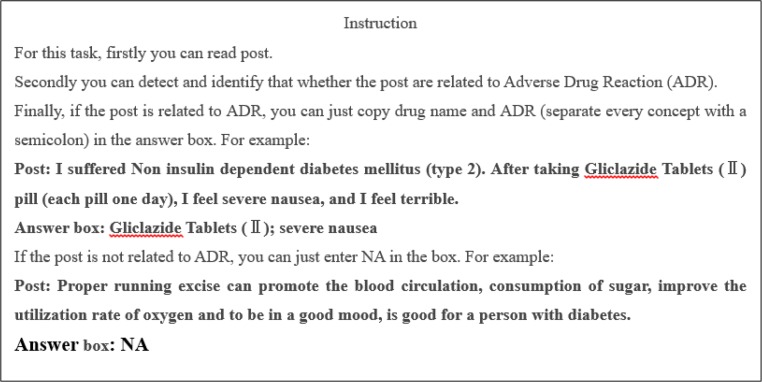
ADR related post detection and identification instruction

We used the instruction to help expert to complete annotate task. The task purpose was to detect and identify that whether the post was related to ADR (Fig. 1).

### Feature set generation

N-gram and Domain feature. N-gram is a kind of language model for text recognition. In N-gram features, the unigram (n=1), bigram (n=2) and trigram (n=3) have been widely used. The N-gram features (n<=3) and domain features were used to represent text. The domain features contained COSTART, SIDER, MedEffect, and CHV (Table 1).

**Table 1:** Definition of feature set variable

***Variable***	***Value***
F1	N-gram and domain features
F2	Sentiment features, n-gram and domain features
F3	Selected features (sentiment features, n-gram and domain features)

Sentiment features. Patients usually gave positive or negative opinions regarding particular drugs in their posts. If the drugs could help them much, they will have a positive attitude towards the drugs. Otherwise, if the drugs have serious side effects, people will express negative feelings about the drugs. These posts usually contained many sentiment words with high sentiment polarity. These sentiment features could be used to measure effectively whether the posts were related to ADRs, thus these sentiment features were helpful to improve identification performance of ADRs. Because SentiWordNet is lexical which can assign sentiment scores to every word (20), our methods introduced SentiWordNet to extract the negativity to be sentiment features.

Feature selection. *χ*^2^ (CHI) statistics can comprehensively consider the possibility between feature and category (21). The CHI statistics can show better stability when the text material gradually increased, so the study used CHI to select feature. CHI statistics can measure the degree of correlation between a word feature and a category. The evaluation function is

$$\chi_{ij}^{2}=\frac{n{\left(n_{11}\times n_{22}-n_{12}\times n_{21}\right)}^{2}}{\left(n_{11}+n_{12})\times (n_{21}+n_{22})\times (n_{11}+n_{21})\times (n_{12}+n_{22}\right)}$$

When $\chi_{ij}^{2}$ denote correlation degree between a word feature i and a category j, n11 is frequency of word i in category j, n12 is frequency of word i in other categories besides j, n21 is frequency of other words besides i in category j, n22 is frequency of other words besides i in other categories besides j.

The experiments were conducted by using different feature sets to compare the effectiveness of different method. The feature set variable had three values. The first value of feature set variable was F1 which contained N-gram and Domain features; the second value of feature set variable was F2 which contained sentiment features, N-gram and Domain feature; the third value was F3 which were the results after F2 feature were selected using CHI.

### Classification and evaluation

We used support vector machine (SVM) as classification technique in our study because SVM can show a good classification performance for large-scale inputs. The tenfold cross-validation was used to evaluate method. In each fold, 90% posts were used to be training set and 10% posts were used to test set. We used the four traditional classification indexes to assess the performance of our study, which were accuracy, precision, recall, and F-measure.

### Ethics and Law

We only used public comments made by user, and we did not use any user identification data and personal information. Therefore, our study cannot raise any ethical or legal concern.

### Statistical analysis

We used pairwise t-tests on accuracy and F-measure by conducting on 50 bootstrap samples to test the function of different feature sets on posts detection. We considered significance level as 0.01.

## Results

### Comparison of different feature sets

The P-value on accuracy was 0.0006. The P-value on F-measure was 0.0005. *P*-value on accuracy was less than 0.01 and *P*-value on F-measure was less than 0.01 when F1 versus F2. Adding sentiment features was a better method than using N-gram and Domain features. The sentiment feature can improve ADR related posts detection performance.

The P-value on accuracy and P-value on F-measure were less than 0.0001 when F2 versus F3. The posts detection effect of using F3 was far better than the effect of using F2.

After compared P-value of the different feature sets, using the selected feature sets from F2 by CHI can significantly enhance posts detection effect.

### Comparison the ADR-related group with the non-ADR group

We considered the F-measure to be performance detection indexes. Each F-measure index value of ADR-related group was higher than the index value of non-ADR group. The performance of ADR-related post detection was better than the performance of non-ADR post detection owing to introducing sentiment feature and using CHI feature selection technique (Table 2).

**Table 2:** F-measure values in the ADR-related group and the non-ADR group

***Feature set*** ***F-measure*** ***Post group***	***F1(%)***	***F2(%)***	***F3(%)***
Non-ADR	75.4	77.5	79.3
ADR-related	76.7	79.0	83.4
Average	76.1	78.3	81.4

We compared the F-measure index value from different feature sets. The F-measure index value of F2 was higher than it of F1, so compared with the traditional post detection method, the method of introducing sentiment feature can improve performance of post-detection; the F-measure index value of F3 was higher than that of F2, so using CHI feature selection technique can also upgrade effect of post-detection.

Besides, we can gradually upgrade performance of ADR-related post detection by using sentiment features and CHI feature selection technique. We can obtain highest F-measure when using F3 feature sets and our method was a very efficient method to detect ADR-related post.

## Discussion

Our method was a very efficient method to detect ADR-related post which used sentiment features and CHI feature selection technique.

After we had checked large-scale post data, generally speaking, the writing of posts related with ADR expressed negative sentiment. Therefore, the negative sentiment features could be used to measure effectively whether the posts were related to ADR, and adding sentiment features could be helpful to improve detection performance of ADR post. Meanwhile, the combination of lots of features brought noise and redundant features. The CHI feature selection technique can reduce the noise and redundant features. Using sentiment features and CHI feature reduction technique can improve detection performance of ADR post in our study. We compared our method with other papers in terms of feature set and result (Table 3).

**Table 3:** Comparison of our method with other studies

***Feature set and Result***	***Reference (22) Reference***	***Reference (9)***	***Reference (8)***	***Reference (5)***	***Our method***
COSTART		√	√		√
SIDER		√		√	√
MedEffect		√			√
CHV	√				√
Sentiment					√
Result	Recall 71.4%	F-measure 73.9%	Precision 72% Recall 86%	F-measure 80%	F-measure 81.4%

The result of our method was better than others. Most of the previous study related to ADR was to extract medical domain features from social media, which included COSTART, SIDER, MedEffect, and CHV. In our study, we tried to incorporate medical domain feature and sentiment feature to detect posts related to ADR. The results have proved it to be effective that introducing sentiment features to detecting post related to ADR.

The potential bias and imprecision was inevitable during the 1814 posts manual annotating process. Despite bias, 1814 posts were considered to be enough to validate extracting system (14). In future study, we will enlarge post labeling number and add more disease category.

In practice, we provided an easy method of obtaining a lot of posts related to ADR and building a medical corpus related to ADR fast. It can save time and improve efficiency in the research related ADR. The detected posts contained much valuable ADR information which was very helpful for the post-marketing drug surveillance, pharmaceutical companies, medical institutions, and patients.

## Conclusion

Using sentiment feature and CHI feature selection technique was an effective method to detect post related to ADR.

## Ethical considerations

The author completely observed and conformed to all ethical guidelines regarding plagiarism, informed consent, misconduct, data fabrication and/or falsification, double publication and/or submission, and redundancy.
